# Supplementary material for: Structure-based discovery of a novel small-molecule inhibitor of TEAD palmitoylation with anticancer activity
Source: Front Oncol. 2022 Nov 29;12:1021823. doi: 10.3389/fonc.2022.1021823 (PMC9745137; doi:10.3389/fonc.2022.1021823)
Supplement: Supplementary file 1 [file DataSheet_1.pdf]

Supporting information

Structure-based discovery of a novel small-molecule inhibitor of TEAD palmitoylation with anticancer activity

Artem Gridnev,Subhajit Maity, Jyoti R. Misra

Department of Biological Sciences

University of Texas at Dallas, Richardson, TX, United States

Correspondence: jyoti.misra@utdallas.edu

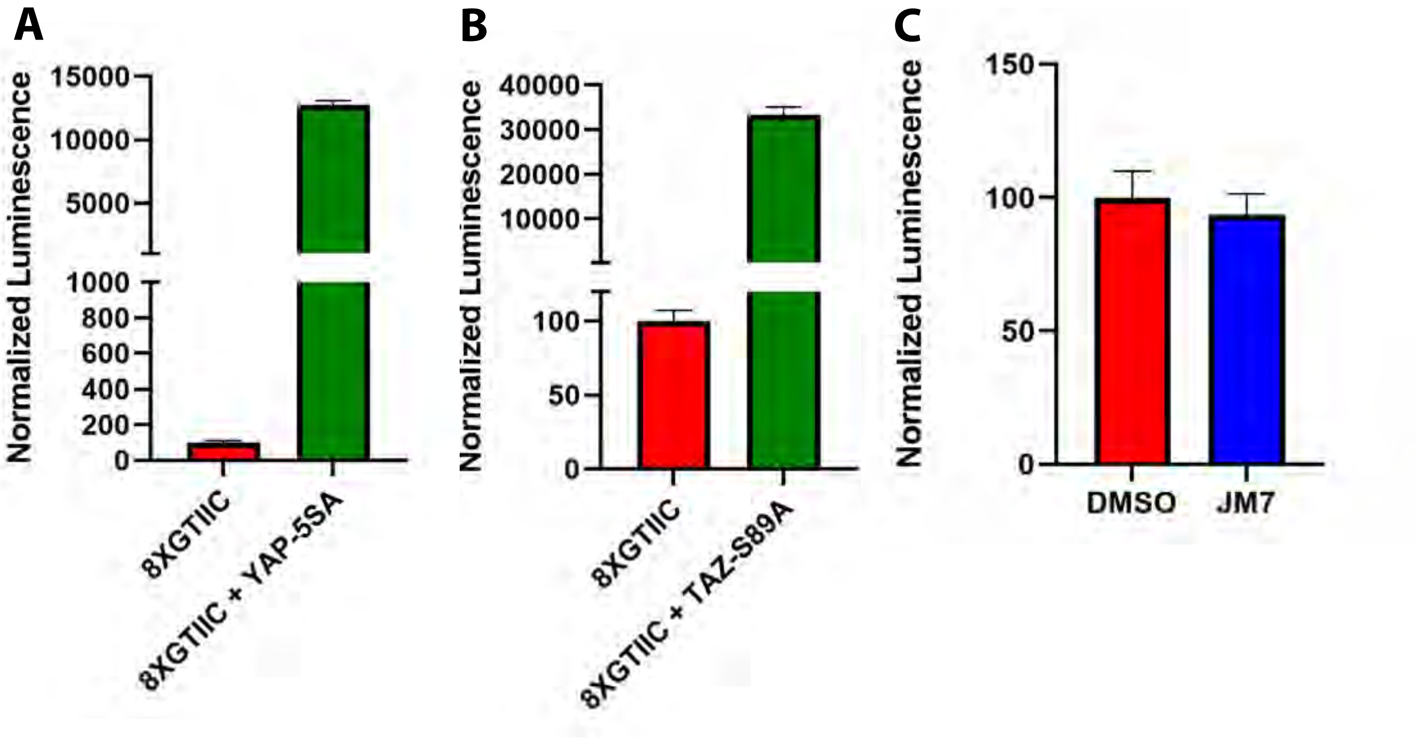

Figure S1. YAP5SA and TAZS89A activates the 8XGTIIC-Luc reporter and JM7 does not inhibit Fluc activity. (A) Histograms showing relative Fluc activity in HEK293 stable cells carrying 8XGTIIC-Fluc reporter transfected with empty vectors or plasmids encoding YAP5SA (A) or TAZS89A (B), indicating very low basal activity of the reporter and hyperactivation of the reporter by YAP5SA and TAZS89A.
(C). Histograms showing relative Fluc activity in HEK293 stable cells carrying SV40 promoter driven Fluc reporter treated with DMSO alone or 2 micromolar JM7, indicating that JM7 does not inhibit Fluc nonspecifically. Error bars indicate standard error of mean (SEM).

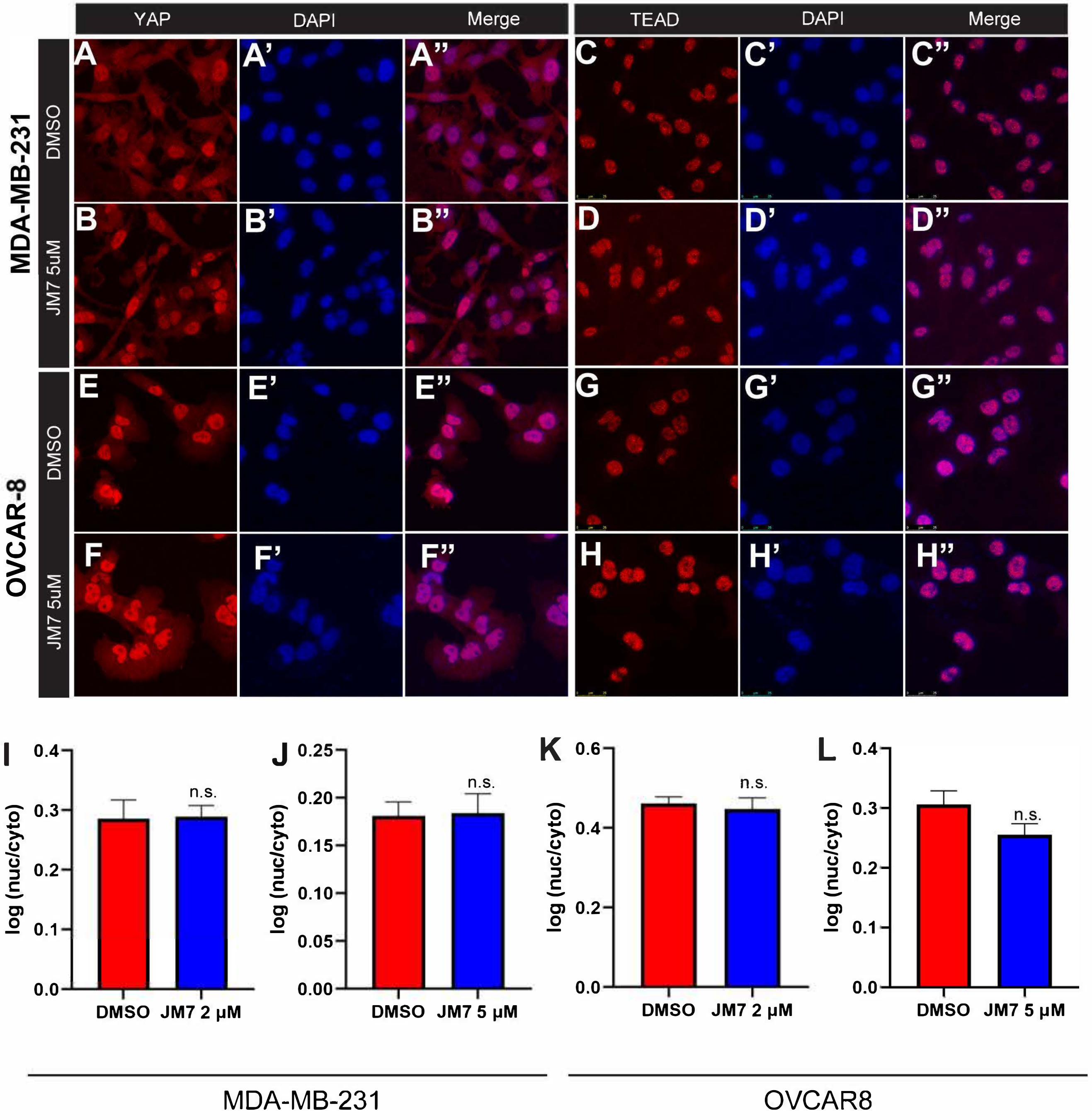

Figure S2. 5 micromolar of JM7 does not affect YAP or TEAD localization.

(A-H") MDA-MB-231 (A-D") and OVCAR-8 (E-H") cells were treated with DMSO or 5 micro molar JM7 and stained with YAP or TEAD antibody, and Hoechst to stain the nuclei showing that JM7 does not seem to affect YAP or TEAD nuclear localization. (I-L) Histograms showing relative nucleocytoplasmic ratio of YAP in MDA-MB-231 (I,J) and OVCAR-8 (K, L) cells treated with DMSO or indicated doses of JM7, showing no significant change in YAP localization. Error bars indicate Standard Error of Mean (SEM) of 10-12 samples. n.s: not significant.

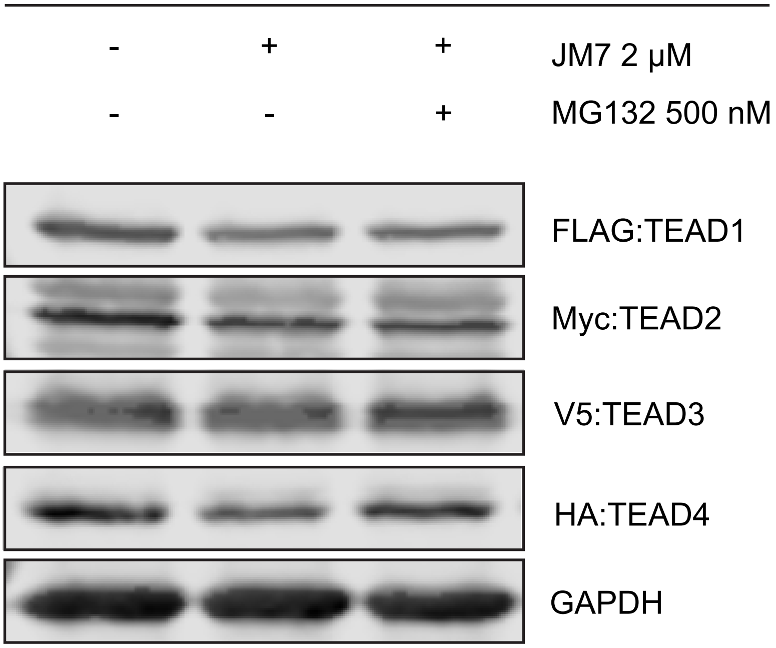

Figure S3. JM7 induces proteosomal degradation of TEAD1-4. HEK-293 cells were transfected with plasmids encoding TEAD1-4 with the indicated epitope tags and treated with DMSO, 2 uM JM7 or 2uM JM7 along with 500nM MG132. The cell lysates were analyzed by Western blotting using antibodies against the respective epitope tags. MG132 reverses JM7 induced degradation of TEAD1-4.

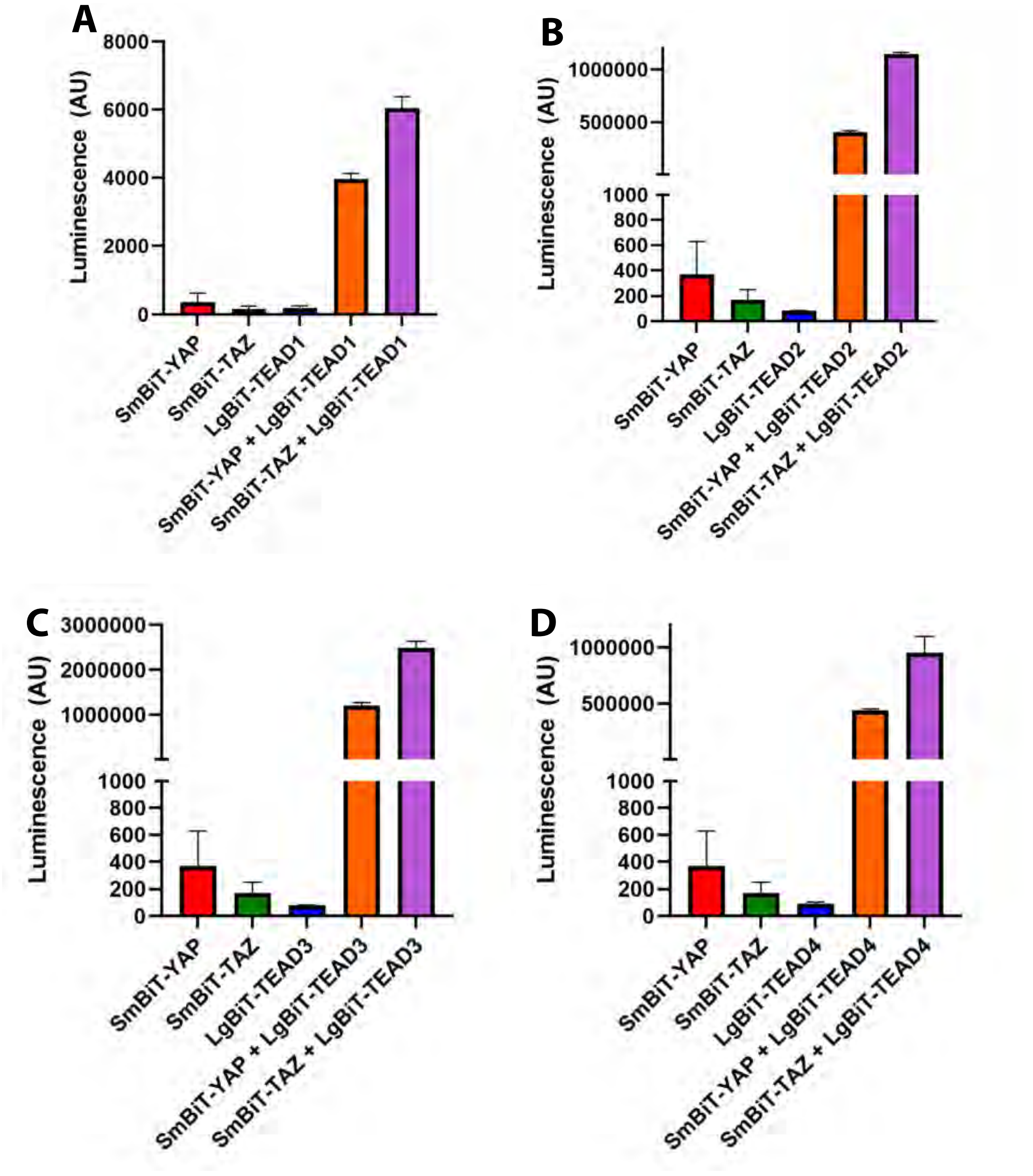

Figure S4. Validation of the nanobit complementation assay.
A-D) Histograms showing relative Nanoluc activity in HEK293 SmBit-Yap (E-H) or Smbit-TAZ. (I-L) together with TEAD1(A), TEAD2 (B), TEAD3 (C) and TEAD4 (D). Error bars indicate Standard Error of Mean.

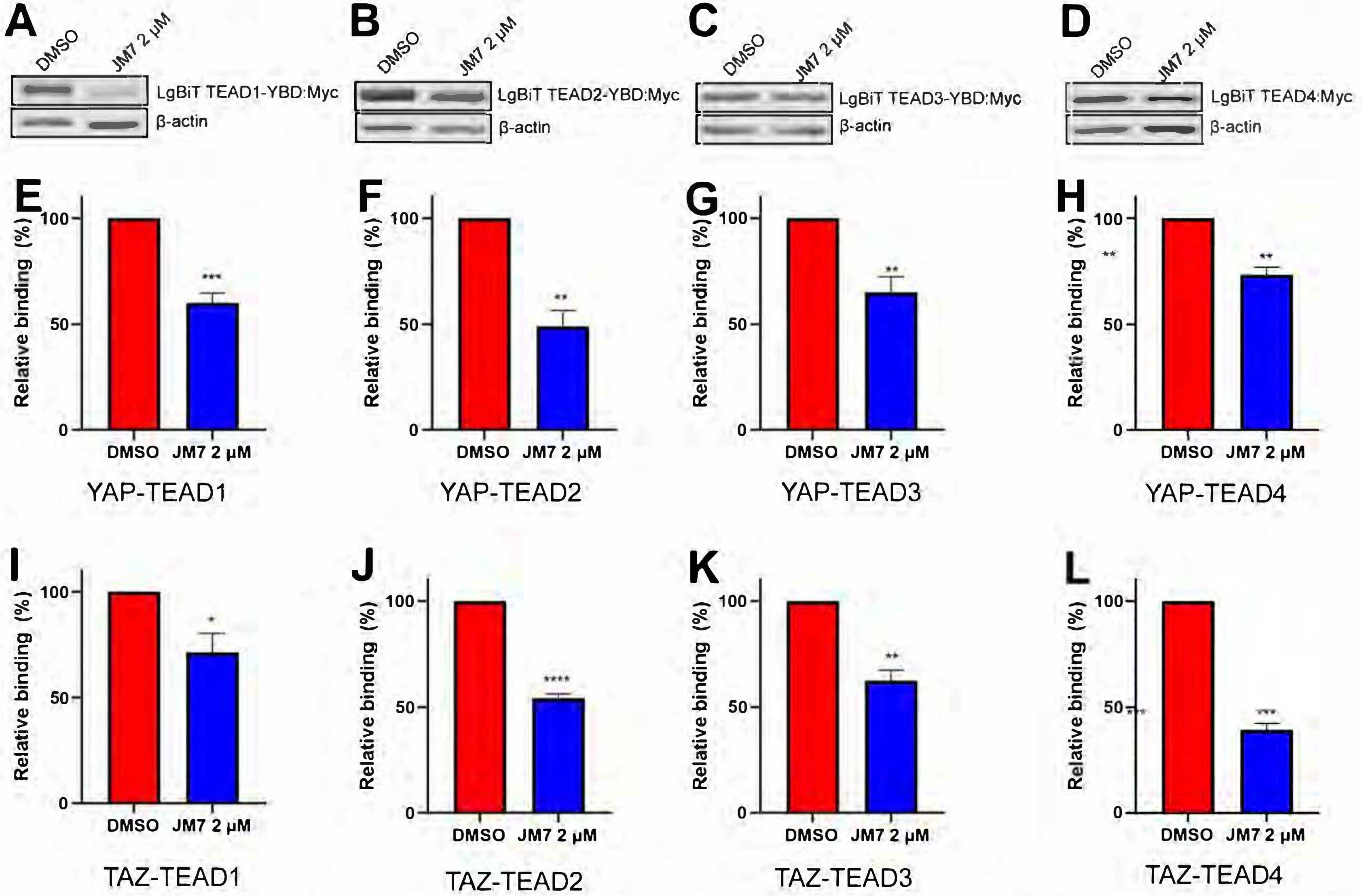

Figure S5. JM7 inhibits nanoBit complementation.(A-D) Western blot showing degradation of LgBit-TEAD1(A), LgBit-TEAD2 (B), LgBit-TEAD3, and LgBit-TEAD1(D) in HEK-293 cells treated with 2uM JM7. (E-L) Histograms showing relative NanoLuc activity in DMSO and JM7 treated HEK293 cells expressing SmBit-Yap (E-H) or Smbit-TAZ (I-L) together with TEAD1 (E, I), TEAD2 (F, J), TEAD3 (G, K) or TEAD4 (H, L). Error bars indicate Standard Error of Mean of 3 replicates. * $p < 0.05$; ** $p<0.005$; *** $p<0.01$; **** $p<0.0005$.

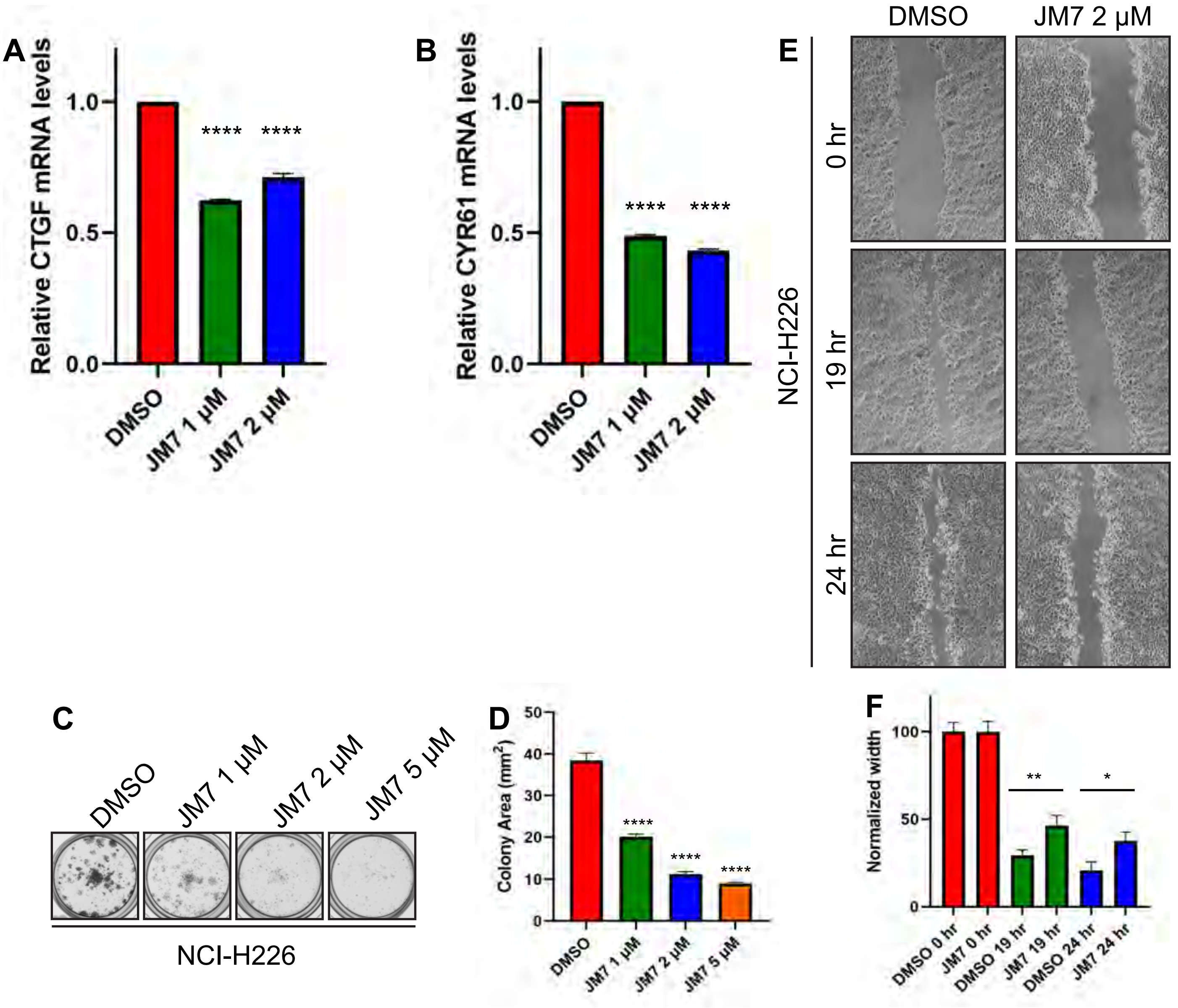

Figure S6. JM7 inhibits YAP target gene expression, colony formation and migration of NF2 mutant mesothelioma cancer cells. (A, B). (A-B) Histograms showing relative expression of CTGF (A) and CYR61 (B) mRNA levels in NCI-H226 cells treated with DMSO or indicated doses of JM7, showing a dose dependent inhibition of these YAP target genes. (C-D) Representative image showing colony formation assay for NCI-H226 cells (C) treated with DMSO or 1, 2 or 5 micromolar JM7 and and quantification of normalized colony area (D). (E, F) Representative images showing wound healing assay for NCI-H226 cells (E) treated with DMSO or 2 micromolar JM7 at different time points. (F) Histogram showing normalized wound width for DMSO and JM7 treated NCI-H226 cells at 0, 19 and 24 hours. * $p < 0.05$; ** $p < 0.005$; **** $p < 0.001$. Error bars indicate Standard Error of Mean (SEM) of three replicates.

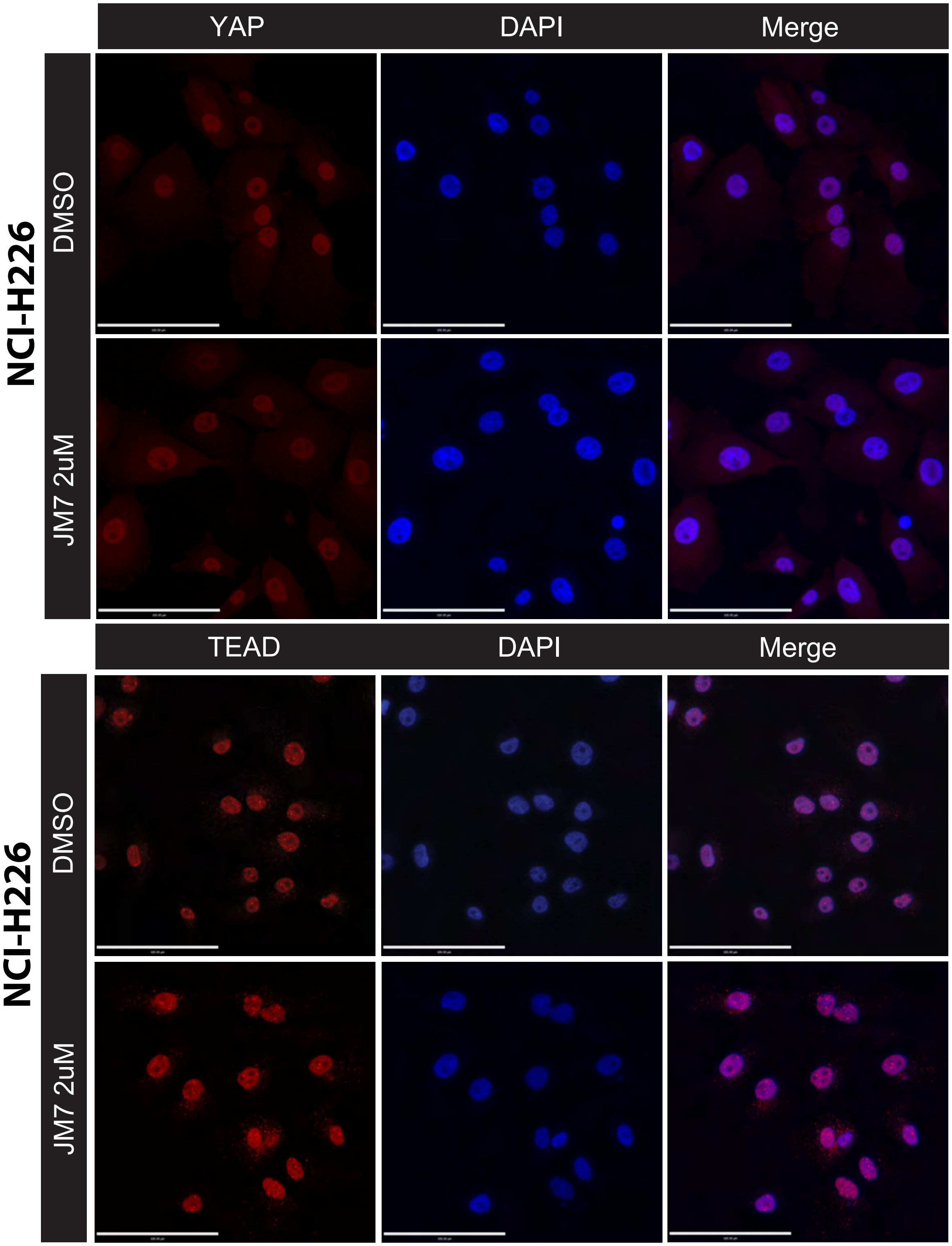

Figure S7. JM7 does not affect YAP and TEAD localization in NF2 mutant mesothelioma cells. NCI-H226 cells were treated with DMSO or 2 micromolar JM7 and stained with YAP or TEAD antibody and Hoechst to stain the nuclei showing that JM7 does not seem to affect YAP or TEAD nuclear localization. Scale bar 100μM.

Supplementary table-1
Predicted physicochemical and pharmacokinetic properties of JM7

**Physicochemical Properties**	
Formula	C21H20N2O6
Molecular weight	396.39 g/mol
Num. heavy atoms	29
Num. aromatic heavy atoms	12
Fraction Csp3	0.24
Num. rotatable bonds	6
Num. H-bond acceptors	6
Num. H-bond donors	2
Molar Refractivity	110.01
Topological Polar Surface Area	95.12 Å²
**Lipophilicity**	
Log Po/w (iLOGP)	3.16
Log Po/w (XLOGP3)	2.29
Log Po/w (WLOGP)	1.56
Log Po/w (MLOGP)	1.74
Log Po/w (SILICOS-IT)	2.75
Consensus Log Po/w	2.3
**Water Solubility**	
Log S (ESOL)	-3.65
Solubility	8.86e-02 mg/ml ; 2.24e-04 mol/l
Class	Soluble
Log S (Ali)	-3.93
Solubility	4.71e-02 mg/ml ; 1.19e-04 mol/l
Class	Soluble
Log S (SILICOS-IT)	-6.04
Solubility	3.60e-04 mg/ml ; 9.08e-07 mol/l
Class	Poorly soluble
**Pharmacokinetics**	
GI absorption	High
BBB permeant	No
P-gp substrate	Yes
CYP1A2 inhibitor	No
CYP2C19 inhibitor	Yes
CYP2C9 inhibitor	Yes
CYP2D6 inhibitor	No
CYP3A4 inhibitor	Yes
Log Kp (skin permeation)	-7.09 cm/s
**Druglikeness**	
Lipinski	Yes; 0 violation
Ghose	Yes
Veber	Yes
Egan	Yes
Muegge	Yes
Bioavailability Score	0.55

Supplementary Table 2

List of oligonucleotides used for molecular cloning in this study.

Oligo name	Sequence (5'-3')
pCDNA3.1-myc-TEAD1 FWD	TAGTCCAGTGTGGTGGAATTCGCCACCATGGAGCAGAAGCTGATCAGCGAGGAGGACCTGATGAGTGACTCTGCAGATAAGCCA
pCDNA3.1 TEAD1 REV	TGCTGGATATCTGCAGAATTCCTAGTCCTTTACAAGCCTGTAAATATGATGTTGTG
pCDNA3.1-myc-TEAD2 FWD	TAGTCCAGTGTGGTGGAATTCGCCACCATGGAGCAGAAGCTGATCAGCGAGGAGGACCTGGGGGAACCCCGGGCT
pCDNA3.1 TEAD2 REV	TGCTGGATATCTGCAGAATTCCTAGTCCCTGACCAGGCGG
pcDNA3.1 LgBiT FWD	TAGTCCAGTGTGGTGGAATTCGCCACCATGGTCTTCACACTCGAAGATTTCGT
pCDNA3.1 LgBiT TEAD1 REV	TGCTGGATATCTGCAGAATTCCTACAGATCCTCTTCTGAGATGAGTTTTTGTTCA
LgBiT REV TEAD2 OH	TGGGGGCAGGGGTGAACCGCTCGAGCCTCC
TEAD2 FWD LgBiT OH	GGAGGCTCGAGCGGTTCACCCCTGCCCCCAC
pCDNA3.1 LgBiT TEAD2 REV	TGCTGGATATCTGCAGAATTCCTACAGATCCTCTTCTGAGATGAGTTTTTGTTCGTCCCTGACCAGGCGG
LgBiT REV TEAD3 OH	ACTAGGGAGTGGTGCACCGCTCGAGCCTCC
TEAD3 FWD LgBiT OH	GGAGGCTCGAGCGGTGCACCACTCCCTAGTGCC
pCDNA3.1 LgBiT TEAD3 REV	TGCTGGATATCTGCAGAATTCCTACAGATCCTCTTCTGAGATGAGTTTTTGTTCATCTTTCACCAGCTTGTACACGT
LgBiT REV TEAD4 OH	GGGCGATGGGGCGGGACCGCTCGAGCCTCC
TEAD4 FWD LgBiT OH	GGAGGCTCGAGCGGTCCCGCCCCATCGC
pCDNA3.1 LgBiT TEAD4 REV	TGCTGGATATCTGCAGAATTCCTACAGATCCTCTTCTGAGATGAGTTTTTGTTCTTCTTTCACCAGCCTGTAGATGTGG
pCDNA3.1 SmBiT FWD	TAGTCCAGTGTGGTGGAATTCGCCACCATGGTGACCGGCTACCG
pCDNA3.1 SmBiT YAP REV	TGCTGGATATCTGCAGAATTCCTACTTGTCGTCATCGTCTTTGTAGTCTACAT
pCDNA3.1 SmBiT TAZ REV	TGCTGGATATCTGCAGAATTCCTACTTGTCGTCATCGTCTTTGTAGTCGT
pCDNA3.1 TAZ FWD	TAGTCCAGTGTGGTGGAATTCATGGCCTACCCATACGATGTTCC
TAZ S89A REV	CAGGGACGCGGGCGAGGCGTGCGAGCGGACATGTTGG
TAZ S89A FWD	CATGTCCGCTCGCACGCCTCGCCCGCGTCCCT
pCDNA3.1 TAZ REV	TGCTGGATATCTGCAGAATTCTTACAGCCAGGTTAGAAAGGGCTC
GAPDH FWD qPCR	GAAGGTCGGAGTCAACGGATT
GAPDH REV qPCR	CGCTCCTGGAAGATGGTGAT
CTGF FWD qPCR	GTTTGGCCCAGACCCAACTA
CTGF REV qPCR	GGCTCTGCTTCTCTAGCCTG
CYR61 FWD qPCR	CAGGACTGTGAAGATGCGGT
CYR61 REV qPCR	GCCTGTAGAAGGGAAACGCT

Supplementary Table-3

List of key resources used in this study.

REAGENT or RESOURCE	SOURCE	IDENTIFIER
**Antibodies**		
YAP (D8H1X) Rabbit mAb	Cell Signaling Technology	14074
Pan-TEAD (D3F7L) Rabbit mAb	Cell Signaling Technology	13295
Myc-Tag (9B11) Mouse mAb	Cell Signaling Technology	2276
Myc-Tag (71D10) Rabbit mAb	Cell Signaling Technology	2278
Pierce Anti c-Myc Agarose	Fisher Scientific	PI20168
Goat anti-Rabbit IgG IRDye 800CW conjugate	LI-COR	925-32211
Goat anti-Mouse IgG IRDye 680RD conjugate	LI-COR	925-68070
Alexa Fluor® 647 AffiniPure Donkey Anti-Rabbit IgG (H+L)	Jackson ImmunoResearch	711-605-152
Bacterial and virus strains		
Mach1 T1^R Chemically Competent *E. coli*	ThermoFisher	C862003
TEAD Luciferase Reporter Lentivirus	BPS Bioscience	79833
**Chemicals, peptides, and recombinant proteins**		
DyLight 680-Conjugated Streptavidin	Rockland Immunochemicals	S000-44
JM7	Vitas-M Laboratory	STK096693
Alkynyl Palmitic Acid	Click Chemistry Tools	1165
Biotin Azide	Click Chemistry Tools	1265
tris (3-hydroxypropyltriazolylmethyl) amine (THPTA)	Click Chemistry Tools	1010
Tris(2-carboxyethyl) phosphine (TCEP) hydrochloride solution	Sigma-Aldrich	646547
Copper (II) sulfate pentahydrate	Sigma-Aldrich	C8027
IGEPAL-CA630	Sigma-Aldrich	I3021
Invitrogen TRIzol Reagent	ThermoFisher	15596026
Applied Biosystems Fast SYBR Green Master Mix	ThermoFisher	4385612
Lipofectamine 3000	ThermoFisher	L3000015
Hoechst 33342	ThermoFisher	H3570
Vectashield mounting medium	Vector Laboratories	H1000
**Critical commercial assays**		
Dual-Luciferase Reporter Assay System	Promega	E1980
Nano-Glo Luciferase Assay System	Promega	N1120
CellTiter-Glo 2.0 Cell Viability Assay	Promega	G9242
ONE-Step Luciferase Assay System	BPS Bioscience	60690
MTT Assay Kit	abcam	ab211091
NEBuilder HiFi DNA Assembly Master Mix	New England BioLabs	E2621
**Experimental models: Cell lines**		
HEK293	ATCC	CRL-1573
MDA-MB-231	ATCC	HTB-26

OVCAR8	NCI	
**Oligonucleotides**		
Please refer to table S1		
**Recombinant DNA**		
pCMX-GAL4-TEAD1	Addgene	33108
pCMX-GAL4-TEAD2	Addgene	33107
pCMX-GAL4-TEAD3	Addgene	33106
pCMX-GAL4-TEAD4	Addgene	33105
pRK5-Myc-TEAD4	Addgene	24638
pCMV-Flag-YAP-5SA/S94A	Addgene	33103
GST-YAP2	Addgene	24637
pCDNA3-HA-TAZ	Addgene	32839
pRL-TK	Promega	E2241
Myc-TEAD3 gBlock	IDT	
pDONR221-TEAD3	DNASU	HsCD00963910
pCDNA3.1-TAZ-S89A	This study	
pCDNA3.1-Myc-TEAD1	This study	
pCDNA3.1-Myc-TEAD2	This study	
pCDNA3.1-Myc-TEAD3	This study	
pCDNA3.1-Myc-TEAD4	This study	
pCDNA3.1-SmBiT-YAP-FLAG	This study	
pCDNA3.1-SmBiT-TAZ-FLAG	This study	
pCDNA3.1-LgBiT-TEAD1-myc	This study	
pCDNA3.1-LgBiT-TEAD2-myc	This study	
pCDNA3.1-LgBiT-TEAD3-myc	This study	
pCDNA3.1-LgBiT-TEAD4-myc	This study	
**Software and algorithms**		
Fiji	NIH	https://imagej.net/Fiji; RRID: SCR_002285
Schrödinger software	Schrödinger Inc	https://www.schrodinger.com/
Prism 9	GraphPad	https://www.graphpad.com/
Volocity	PerkinElmer	https://www.perkinelmer.com/
LAS X Life Science Microscope Software	Leica	https://www.leica-microsystems.com/products/microscope-software/p/leica-las-x-ls/
